# Airflow in the Human Nasal Passage and Sinuses of Chronic Rhinosinusitis Subjects

**DOI:** 10.1371/journal.pone.0156379

**Published:** 2016-06-01

**Authors:** Haribalan Kumar, Ravi Jain, Richard G. Douglas, Merryn H. Tawhai

**Affiliations:** 1 Auckland Bioengineering Institute, The University of Auckland, Auckland, New Zealand; 2 Department of surgery, The University of Auckland, Auckland, New Zealand; Technion - Israel Institute of Technology, ISRAEL

## Abstract

Endoscopic surgery is performed on patients with chronic inflammatory disease of the paranasal sinuses to improve sinus ventilation. Little is known about how sinus surgery affects sinonasal airflow. In this study nasal passage geometry was reconstructed from computed tomographic imaging from healthy normal, pre-operative, and post-operative subjects. Transient air flow through the nasal passage during calm breathing was simulated. Subject-specific differences in ventilation of the nasal passage were observed. Velocity magnitude at ostium was different between left and right airway. In FESS, airflow in post-surgical subjects, airflow at the maxillary sinus ostium was upto ten times higher during inspiration. In a Lothrop procedure, airflow at the frontal sinus ostium can be upto four times higher during inspiration. In both post-operative subjects, airflow at ostium was not quasi-steady. The subject-specific effect (of surgery) on sinonasal interaction evaluated through airflow simulations may have important consequences for pre- and post-surgical assessment and surgical planning, and design for improvement of the delivery efficiency of nasal therapeutics.

## Introduction

Chronic Rhinosinusitis (CRS) is a persistent inflammatory disease of the paranasal sinuses that is characterized by clinical symptoms that include a blocked nasal airway, mucus discharge, facial pain, headaches and anosmia [1, 2]. Functional endoscopic sinus surgery (FESS) is performed on patients who fail to improve following medical therapies such as antibiotics and corticosteroids (both systemic and topical nasal sprays). In sinus surgery, the goals are to open the obstructed sinus openings (ostia), to improve sinus ventilation and to restore mucociliary clearance. After initial surgery, a number of patients may continue to have ongoing symptoms and recalcitrant disease for which a more extensive operation such as the Modified Endoscopic Lothrop procedure (MELP) is performed [3–5]. The MELP procedure differs from standard frontal sinus dissection because both the frontal beak that narrows the frontal ostia, and the adjacent upper part of the nasal septum and frontal intersinus septum are removed, creating a single large common drainage pathway for both frontal sinuses. Current understanding of the relationship between nasal geometry (pre- and post-operative) and sinus ventilation is poor; and despite surgical intervention, efficient topical distribution of therapeutic drugs remains a significant challenge. Simulating nasal airflow in this complex patient group will improve our understanding of how surgical strategies affect post-surgical sinus ventilation, as well as providing new understanding for how drug delivery treatments and devices [6–10] can be designed to target delivery to the sinuses.

Nasal passage is connected to sinus air pockets through an opening called ostia. Airflow in the human nasal cavity has been extensively studied using fluid dynamic simulations. We refer the reader to [11] and references there on. A number of studies have simulated airflow in both nasal passage and the sinuses [10, 12–24]. Xiong et al [12] simulated nasal airflow at 21 L/min in a normal healthy subject and found very little flow between the nasal passage and the sinuses. At the frontal sinus ostium they observed a limited flow rate of 0.014mL/s during inspiration and 0.018 mL/s during expiration. Zhu et al. [20] evaluated post-surgical airways after uncinectomy and bilateral inferior turbinate reduction and noticed that the surgery that aimed to affect flow partitioning also increased sinus ventilation in only one respiratory phase. The effects of surgery on altering nasal airflow is a complex realm and are not completely understood. Also, these studies do not sufficiently describe airflow in the sinus.

This study describes airflow in the nasal passage and sinuses using fluid dynamic simulations. Specifically, airflow in pre-operative and post-operative CRS subject is investigated. FESS in CRS patients is known to increase nasal airway patency, however although this leads to reduced nasal resistance, the role of surgery in altering exchange of air between the sinus and nasal passages is not clear. Transient airflow is simulated in a healthy normal subject, a pre-operative subject with CRS, the same subject post-operatively after a standard FESS procedure, and a post-operative subject after a Lothrop procedure. Particular focus is given to describing airflow at the openings to the frontal and maxillary sinuses.

## Methods

Computed tomographic (CT) imaging of human head in a normal, pre- and post-operative subject were obtained following approval from New Zealand Health and Disability Ethics committee. Written consent was obtained from all patients. Comprehensive information sheet and signed consent was obtained from all patients. Information sheets, consent forms and procedures were all reviewed and approved by the ethics committee. Images were acquired using a 16-slice CT scanner, with 1 mm axial slices and resolution of 0.35 mm/pixel. CT scans from three subjects were analysed:

1. Healthy normal subject (refereed henceforth as subject 1)
2. pre-operative subject with CRS (referred henceforth as subject 2a)
3. post-operative scan of subject in (ii) after a standard FESS procedure (referred henceforth as subject 2b). In this case, the surgery procedure was a full-house FESS including ethmoidectomy, uncinectomy, inferior turbinectomy and maxillary antrostomy [25–28].
4. post-operative scan of a subject after a Lothrop procedure referred here as drill-out subject (referred henceforth as subject-3)

The nasal airways were segmented using *MATLAB* (The Mathworks, Natlick, MA, USA). The masks were converted into a triangulated surface using *ITK-Snap* [29]. The resulting STL file was edited for any non-physical features and further processed using *MeshLab*. Unstructured mesh was created in *ICEM CFD* (ANSYS Inc., USA). In each case, mesh dependency was examined using steady state simulation. Velocity and wall shear was compared. In each case, average velocity was compared at three coronal planes. For the normal subject, three meshes with approximately 2 million, 5 million and 10 million elements were generated. In the three coronal planes, average wall shear successively changed by 7% and 4%, for the two steps of mesh refinement. Average velocity changed by 4% and 1% respectively. For the post-operative subject-2b, three tetrahedral-prismatic meshes with 3 million, 7 million and 12 million elements were generated. On the three coronal planes, average wall shear changed successively by 10% and 7% with mesh refinement and average velocity changed by 5% and 1%, respectively. For the drill-out subject-3, three tetrahedral-prismatic meshes with 2.5 million, 6.6 million and 13 million elements. Average wall shear reduced by 15% and 4% with mesh refinement while average velocity by 11% and 5%. For laminar flows, hybrid meshes [30] have been shown previously to yield higher root-mean squared indices with refinement due to mesh not aligning with predominant flow. Hence we used only average wall shear and velocity. Within acceptable values of velocity, the medium mesh in each of the above case was further refined in nasal vestibule and sinus ostium. The final mesh consisted of 8 to 10 million (except for pre-operative airway with 5.7 million tetrahedral-prismatic elements) elements and three prismatic layers with total height of ~0.3 mm from wall. Airflow during rhythmic breathing was simulated using *ANSYS CFX* 16.0 (ANSYS Inc., USA), a high-performance fluid dynamic solution package. Simulation of quiet breathing was performed at peak flow rate 12 L/min specified at outlet of our bilateral nasal airway models. A laminar flow solver using a second order backward Euler scheme for transient terms was used. To provide a natural boundary condition between the nose and its surrounding structures, a mask-like surface was attached to the nose. This is referred to as face boundary [31]. A zero pressure opening-type boundary condition is imposed on this surface. A straight tube was attached to nasopharyngeal end of the geometry. To achieve fully developed flow profile in non-circular tubes, the following steps were performed. For each case, a separate quasi-steady flow was first simulated with an inlet flow rate specified on the face boundary and zero pressure on the tracheal outlet boundary. The velocity ***u***_*p*_*(x*,*y*,*z)* = (*u*_*P*_,*v*_*P*_,*w*_*P*_) that resulted from this simulation, at each mesh point on this outlet boundary was stored. For steady-state simulations, this velocity (= ***u***_*P*_*(x*,*y*,*z))* was specified as boundary condition at the outlet. For all transient simulations, a time-dependent velocity (= ***u***_*P*_*(x*,*y*,z)*sin(2πt/T)) was specified at the outlet boundary, where t is time and T = 4 seconds is the breathing period. For all the cases, Reynolds number and Womersley number was computed. Reynolds number Re = UD/ν where U was maximum speed in the nasal valve region. Hydraulic diameter ‘D’ (= 4*area/perimeter) was computed at nasal valve and nasopharynx. If ‘ω’ is the imposed frequency, ‘ν‘ is kinematic viscosity, then Womersley number, α = (D/2)√(ω/ν) was found to be in range of 1.0 to 1.6 for cases investigated here. For post-operative situations with very large ostia, entrainment between nasal passage and cavity may become significant resulting in transient flow and the theoretical limit of α = 1 may not be valid [32]. To avoid such ambiguities, an unsteady solver is adopted here. The simulations in this study were run upto 3 cycles. Adaptive time stepping was chosen with maximum Courant number of 6 and a minimum time step of 3x10^-4^ sec in subject-1, 1.6x10^-4^ sec in subject-2b and 1.2x10^-4^ sec in subject-3. Velocity at three different locations were monitored. Root mean squared error of velocity was within 0.1% between last two cycles.

## Results

### Nasal and Sinus Geometry

**Fig 1:**
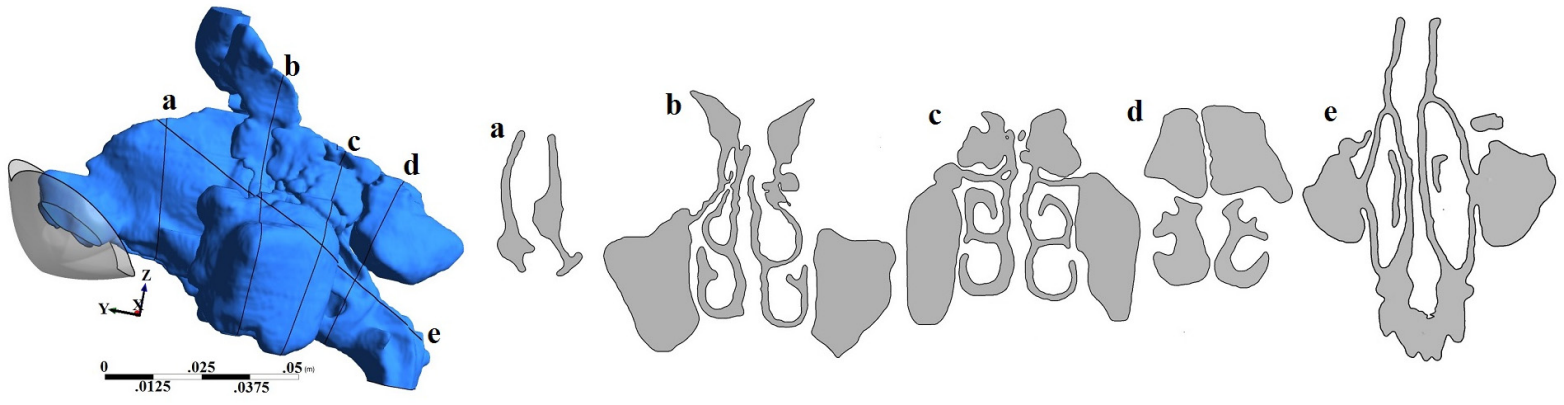
3D nasal cavity geometry of a healthy normal subject and cross-sectional views of the nasal airway. Figures drawn to scale.

**Fig 2:**
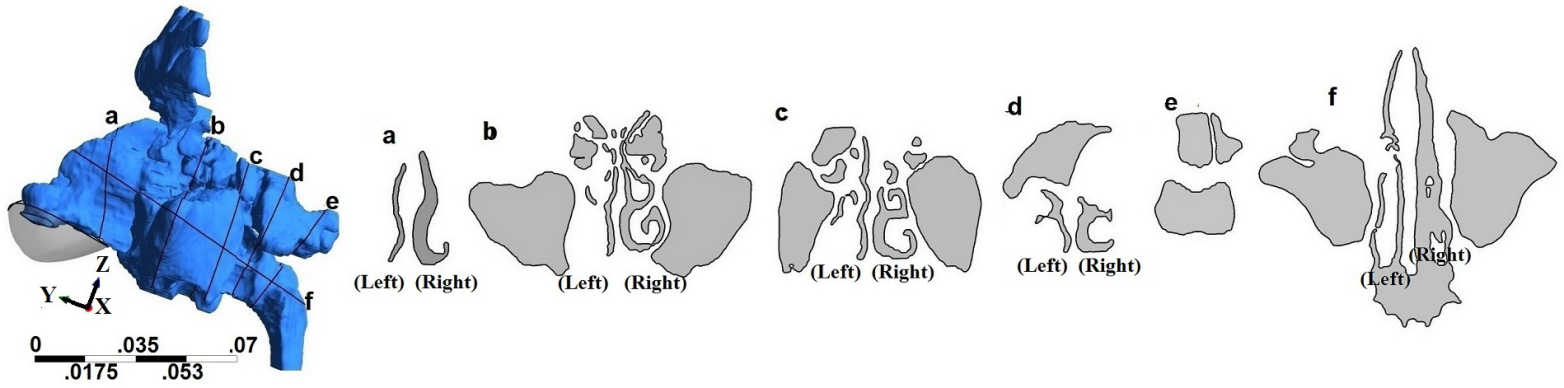
3D nasal cavity geometry of a pre-operative subject and cross-sectional views of the nasal airway. Figure drawn to scale.

**Fig 3:**
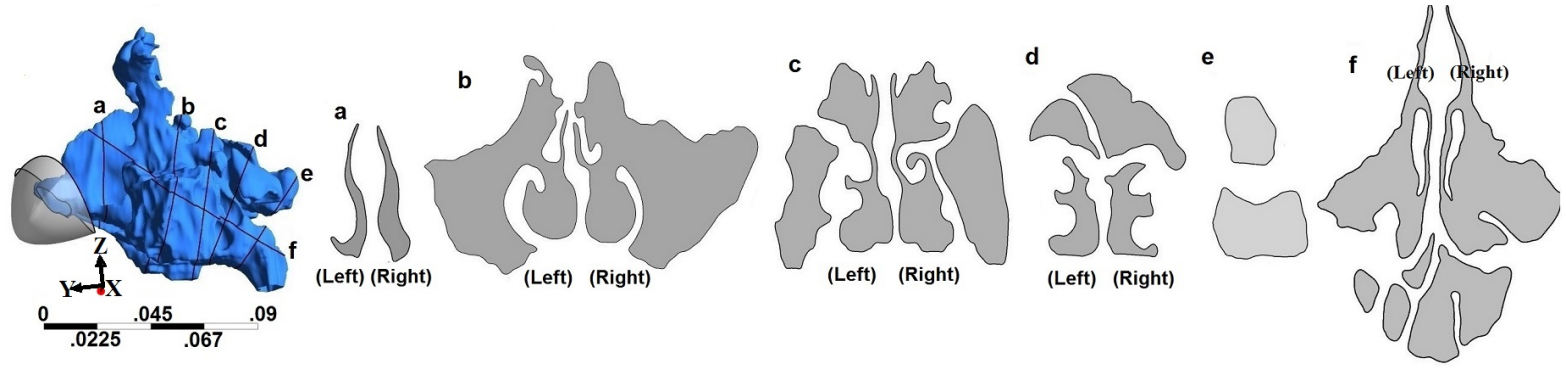
3D nasal cavity geometry of a post-operative subject after regular functional endoscopic surgery and cross-sectional views of the nasal airway. Figures are to scale.

**Fig 4:**
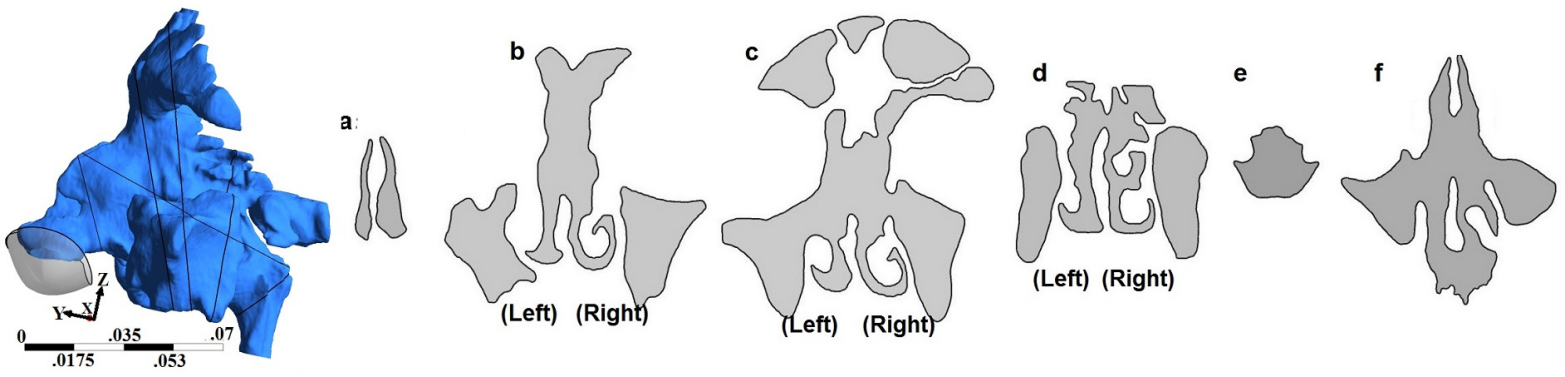
3D nasal cavity geometry of the drill-out subject and cross-sectional views of the nasal airway. Figure drawn to scale.

Geometry and sectional views for all four geometries are shown in Figs 1–4. Reconstructed 3D geometry of the nasal cavity from Subject 1 is shown in Fig 1. Sectional views are shown to scale in Fig 1(b). Slice 5 is an oblique plane that cuts through the maxillary ostia. In Fig 1, maxillary and frontal sinuses are visible in Section-b, maxillary and ethomoidal sinuses in Section-c and sphenoidal sinuses in Section-d. Sections from subject 2a, are shown in Fig 2. The pre-operative airway was the most difficult to segment due to ambiguity of the boundaries of the thin bony septations that were not clearly resolved by the scanner. Both the frontal and maxillary sinuses were found to be completely disconnected from the nasal passages. After surgery, the nasal airway patency increased (Fig 3). Both frontal and maxillary recesses are wide, and opened to the nasal passage. Nasal cross-sections from subject 3 are shown in Fig 4. The common airway in Section-b is due to drilling of the bone through the septum that establishes a common drainage pathway from both frontal sinuses. The shape of vestibule in subject-3 was distinct and resembled a notched phenotype [33] as seen in some subjects.

Table 1 report measurements from the 3D nasal airway. The total nasal volume includes all sinuses and the main nasal passage and for consistent measurement, the geometry up to slightly posterior to the nasal choana (where the left and right nasal airways converge forming the nasopharynx) was used. The overall surface-to-volume ratio of subject 2A was 4.69 cm^-1^, 28% greater than subject 2B. Differences were noted between subjects in the nasal valve and ostium dimensions. Measurements of left and right maxillary ostia sectional area, sinus volumes, frontal ostia sectional area and frontal sinus volumes are reported in Table 2. The entrances to both maxillary and frontal sinuses in subject 2A were completely dissected. The postoperative geometry had a relatively large maxillary ostium as a result of the maxillary antrostromy.

**Table 1:** Measurements related to nasal passage from the three-dimensional geometry for subjects used in this study.

Subject	Nasal wall area (cm^2^)	Nasal cavity volume (cm^3^)	valve area (left, right) (mm^2^)	Valve perimeter (mm)	outlet area (mm^2^)
**Normal**	421.5	85.3	(76,86)	(42, 38)	206
**Pre-op**	467.1	99.5	(98,83)	(41,39)	147
**Postop**	446.5	121.4	(97,84)	(41,39)	149
**Drill-out**	407.5	118.5	(46,112)	(31, 46)	166.4

**Table 2:** Measurements related to sinuses from the three-dimensional geometry for subjects used in this study.

Subject	Maxillary volume, cm^3^ (left+right)	Frontal sinus volume, cm^3^ (left+right)	Maxillary ostium area, mm^2^ (left,right)	Frontal ostium area, mm^2^ (left+right)
Normal	29.9	7.9	(62,65)	166
Pre-op	47.4	13.7	(0,0)	10
Postop	48.4	8.6	(270,180)	102
Drill-out	39.2	24.7	(59,130)	175

### Validation

**Fig 5:**
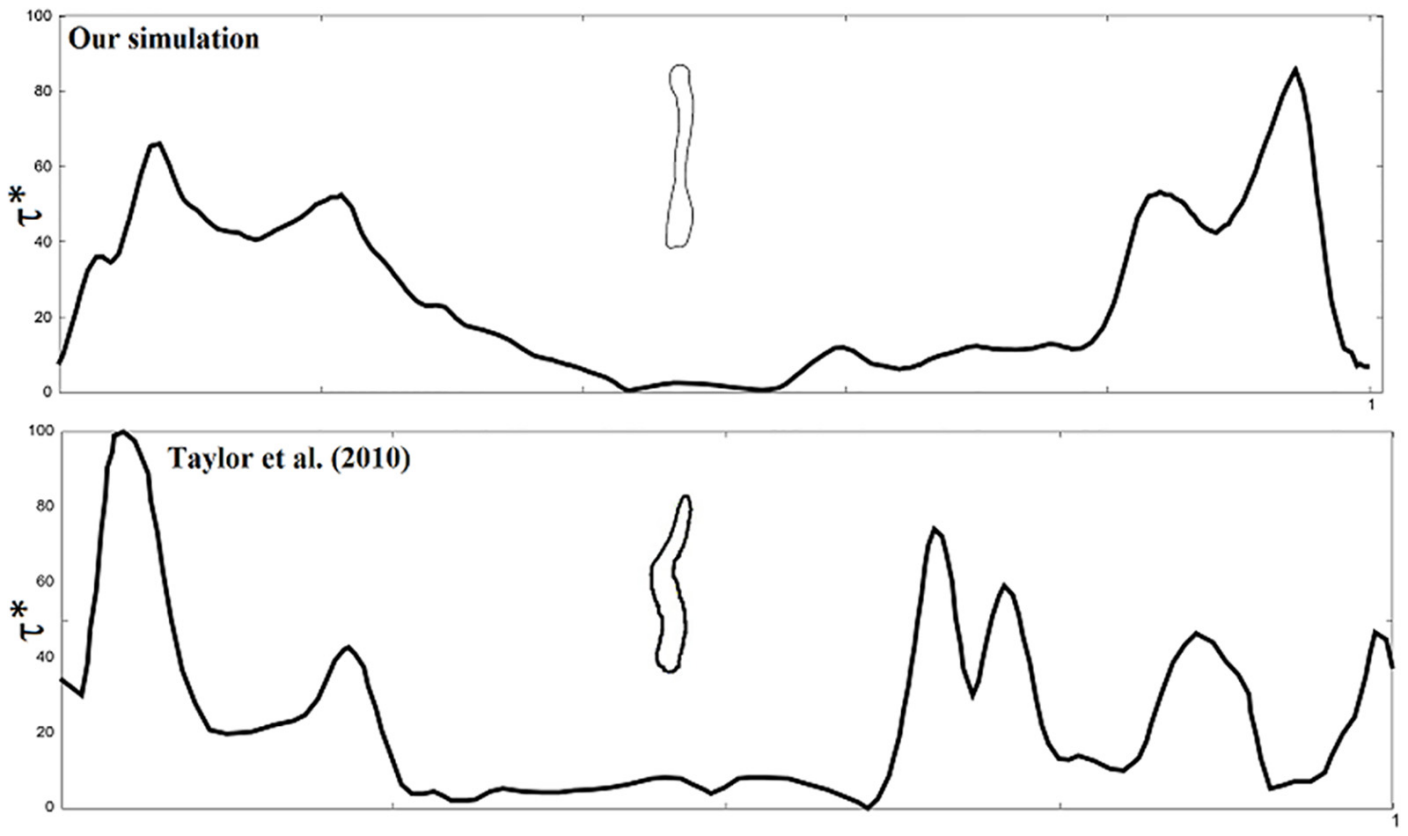
Plot of normalized wall shear stress (τ*) along perimeter of a section in the healthy normal subject. (Comparison with Taylor et al. (2010). Permission license number 3853890090976 obtained from The Royal Society and Copyright Clearance Center. Wall shear is normalized using μU_max_/h_V_ where μ is the dynamic viscosity, U_max_ is the velocity in Table 3 and h_V_ is the hydraulic diameter based on left airway valve area from Table 1.

Nasopharyngeal pressure drop is sensitive to nasal valve area which is generally the minimum cross-sectional area in the nasal passage. The results of maximum pressure drop for subjects in this study are consistent with the nasal valve area. For example, nasal valve area of subject 1 is smaller than case 2B and hence showed greater pressure drop. Taylor et al reported a unilateral airway pressure drop of 1.7 Pa and 8.7 Pa (in two subjects with valve area 95.6 mm^2^ and 40.2 mm^2^, respectively) at 6 L/min [31]. In a separate simulation, we imposed inspiratory flow at a constant rate of 12 L/min using our bilateral nasal airway. In subject 1 with corresponding (left, right) nasal valve region of (76 mm^2^, 86mm^2^), we observed overall inspiratory pressure drop of 5.4 Pa. To compare with Taylor *et al* we plotted normalized wall shear along the perimeter of a section of the nasal airway. Wall shear was normalized using μU/D_V_ where μ is the dynamic viscosity of air, U is the average velocity and D_V_ is the hydraulic diameter based on airway valve area from Table 1. Normalized wall shear is plotted in Fig 5 against normalized distance. Two dominant peaks were observed. Maximum normalized wall shear was about 88 while Taylor et al. observed up to 100 in one subject. The results agree qualitatively although differences may arise due to geometry.

### Nasal resistance

**Fig 6:**
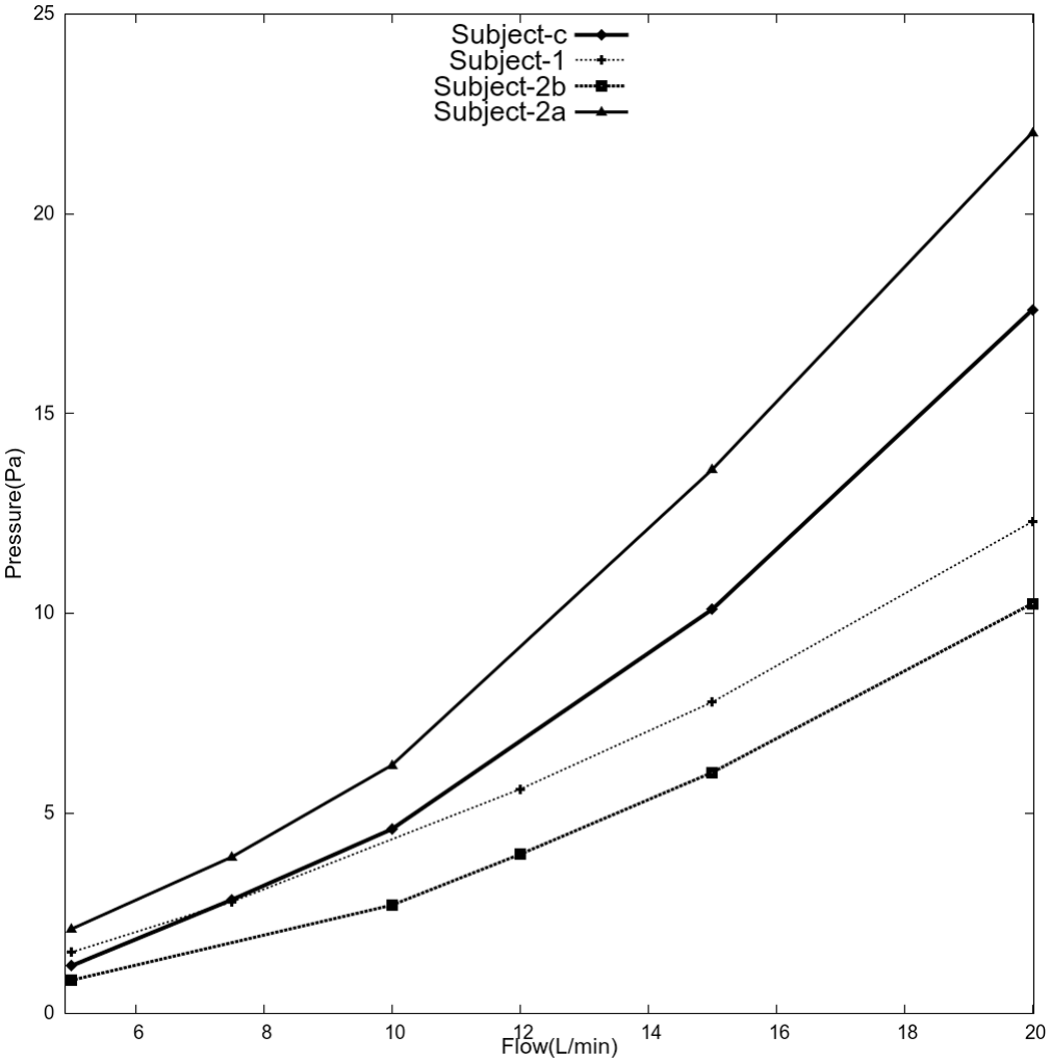
Flow resistance from steady simulations.

Steady flows between 5 L/min and 20 L/min were simulated to obtain inspiratory flow resistance. Resistance informs about patency of airway and is dependent on the minimal cross-sectional area. Resistance curves provide easier and quantitative comparison of nasal anatomy of different subjects. For each flow rate, resulting pressure drop was observed at the nasopharynx in Fig 6. As expected, pressure drop increased with flow rate and was highest in subject 2A. Nasopharyngeal pressure reached to about 10 Pa at 20 L/min in subject-2b compared to about 22 Pa in its pre-operative state subject-2a. For subject-3, steady state solution could not be reached beyond 10 L/min. In these cases, average pressure at the nasopharynx fluctuated within 0.1 Pascals and hence a time-averaged solution was computed. For example, inspiratory pressure was about 17.6 Pascals at 20L/min.

## Airflow Characteristics

**Fig 7:**
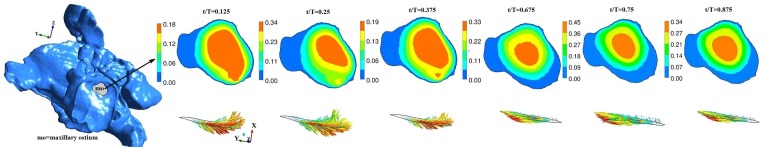
Maxillary ostium and time-evolution of velocity magnitude and velocity vector for normal healthy subject-1. Figure not to scale.

**Fig 8:**
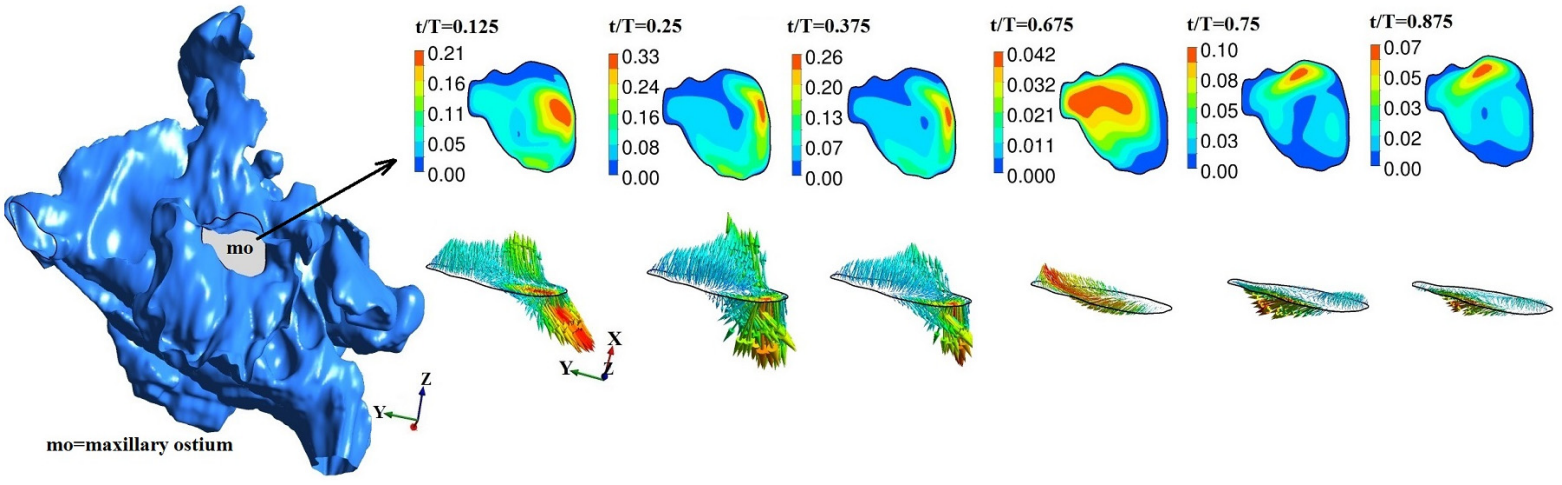
Maxillary ostium and time-evolution of velocity magnitude and velocity vector for subject-2b. Figure not to scale.

**Fig 9:**
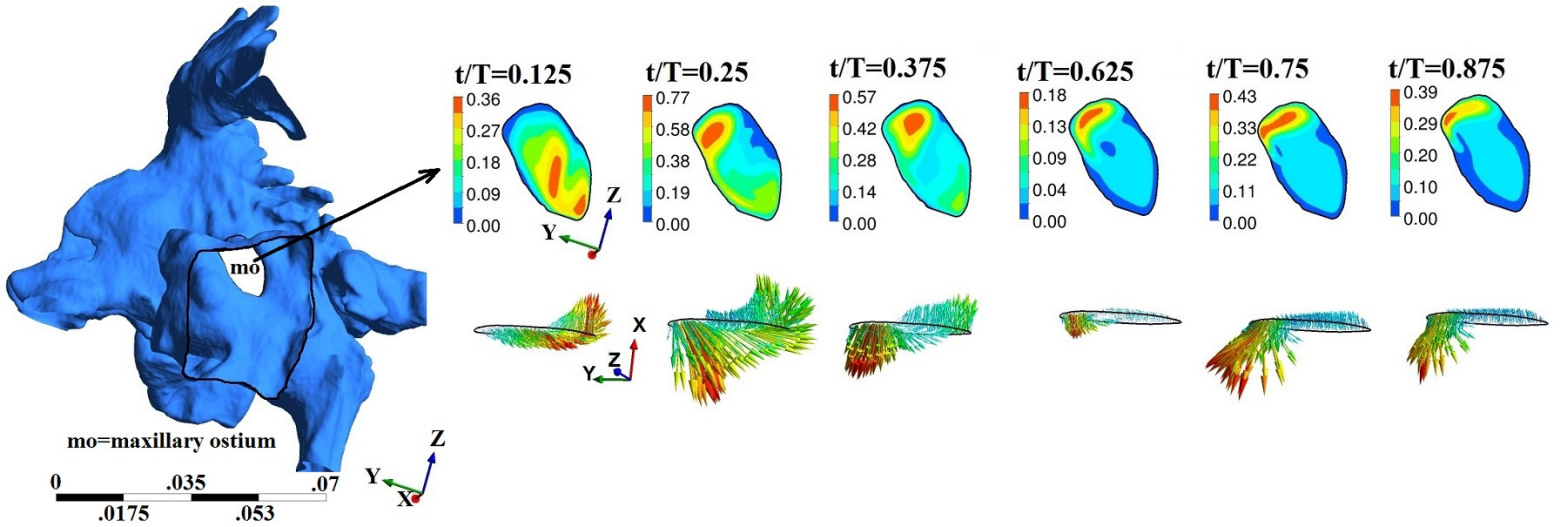
Maxillary ostium and time-evolution of velocity magnitude and velocity vector for subject-3. Figure not to scale.

**Fig 10:**
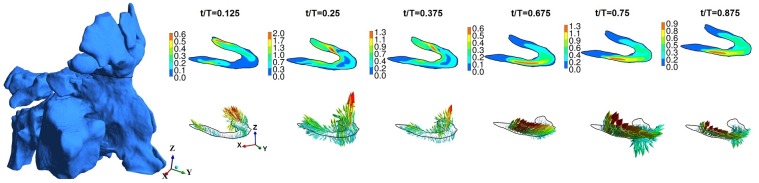
Frontal ostium and time-evolution of velocity magnitude and velocity vector for subject-4. Figure not to scale.

**Fig 11:**
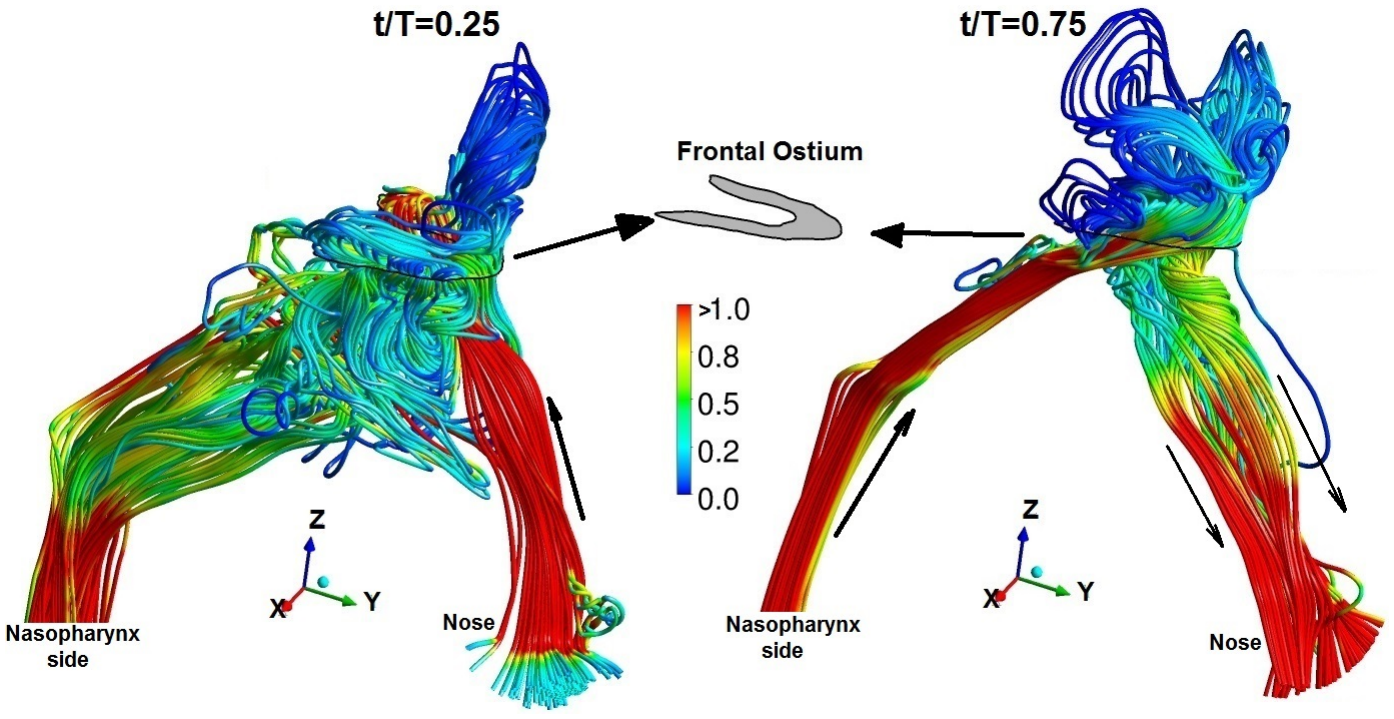
Instantaneous streamline plot at peak inspiration (t/T = 0.25) and peak expiration (t/T = 0.75) near frontal ostium for the drill-out subject.

A total of four transient airflow simulations cases were conducted. Nasopharyngeal pressure, flow at maxillary and frontal sinus ostium, are reported in Figs 7–11. The results show the following similarities in airflow. Because of the face boundary condition at the nose, air was drawn into the nose (during inspiration) from the surface of the face inlet as seen from the streamlines in Fig 7. During expiration, streamlines out of the nose were mostly straight, narrow ending on the face boundary. The maximum velocity was observed near and slightly past the nasal valve. Flow within the maxillary and frontal sinuses recirculates creating complex vortical flows. The geometry of drill-out subject-3 was particularly different (from other airways used in this study) as the left airway nasal valve was 60% smaller than right side. As the nasal vestibule was notched, airflow through left airway was directed towards the middle and inferior meatus while flow through the right airway was predominantly directed towards the frontal ostium.

In addition to these common characteristics, quantitative and qualitative differences were observed. These differences may be attributed to individual anatomy as seen in Table 3. For subject-1, average nasopharyngeal pressure varied between -5.5 Pa (at t/T = 0.25) and 5.24 Pa (at t/T = 0.75). In subject-2A, average nasopharyngeal pressure varied between -9.3 Pa (at t/T = 0.25) and 7.4 Pa (at t/T = 0.75): 25% higher during inspiration. In subject-2b, average nasopharyngeal pressure varied between -4 Pa (at t/T = 0.25) to 2.4 Pa (at t/T = 0.75): 50% higher during inspiration. In subject-3, average nasopharyngeal pressure changed between -6.7 Pa (at t/T = 0.25) to 4.5 Pa (at t/T = 0.75): 50% greater during inspiration. Hence pressure drop was noted to be significantly greater during inspiration then expiration in both subjects 2b and 3.

**Table 3:** Results of nasopharyngeal pressure and airflow speed.

Subject	Nasopharyngeal pressure drop (Pa)		Maximum airflow speed in entire domain, U_max_	Maximum nasopharynx velocity (m/s)	Reynolds number DU_max_/ν	
	t/T = 0.25	t/T = 0.75			Left valve	Nasophyarynx
Normal	5.4	5.24	2.98	1.6	1296	1595
Pre-op	9.3	7.4	3.5	2.5	2090	1980
Postop	4	2.4	2.3	2.0	1360	1587
Drill-out	6.7	4.5	3.2	1.5	1180	1150

The maxillary and frontal ostia in general constitutes a volume that connects the nasal passage and the sinuses. For visualization, we carefully extracted a cross-section of the ostium region and report maximum and average velocity at this plane. Streamlines near the maxillary sinus and time history of velocity at selected coordinates in the ostium are included as supplementary material (S1–S3 Figs).

At the plane of ostium, flow rate (area integral of **u**.**n**, **u** is velocity, **n** is normal) was computed with normal vector pointing into the ostium. Typically it may be expected that only a small fraction of air from nasal passage may enter the sinus. The instantaneous flow at ostium changes sign indicating inflow or outflow. For subject-1, inflows and outflows of upto 6ml/min were observed. For subject-2b, upto 21 ml/min inflow was observed at maxillary ostium. For subject-3, inflow and outflow upto 6 ml/min inflow was observed at maxillary ostium and only inflow upto 8 ml/min at the frontal ostium. Overall, the instantaneous flow was at ostium was in the order of ml/min. For further analysis, velocity was studied. Figs 7–9 shows time evolution of velocity at three times (t/T = 0.125, t/T = 0.25 and t/T = 0.375) during inspiration and three times (t/T = 0.675, t/T = 0.75 and t/T = 0.375) during expiration in the left maxillary ostium. The left ostium has been shown in grey shade. Time history of velocity are plotted to observed oscillatory flow response maxillary ostium (points M1 and M2 in supplementary material S4 Fig). Although magnitude changes, flow at maxillary ostium was largely quasi-steady for subject 1 as seen from Fig 7. Location of peak velocity at ostium does not change during breathing cycle. While in subjects 2b and 3, location of peak velocity shifts during breathing cycle due to local transient effects. For example, compare velocity contours at the ostium at t/T = 0.125 and t/T = 0.25; Also compare t/T = 0.675 and t/T = 0.75. Hence surgery has shifted the location of peak velocity at the maxillary ostium. Velocity contour and vector are plotted at frontal ostium in Subject-3. Location of peak velocity shifts between inspiration and expiration. Also note the velocity contours are different between t/T = 0.125 and t/T = 0.25 indicative of transient behavior. Hence for subject-3, flow is overall transient as evident from streamline plot and time history of velocity magnitude at ostia.

For subject-1, maximum velocity at the left and right maxillary ostium were 0.34 m/s and 0.82 m/s at t/T = 0.25; 0.45 m/s and 0.96 m/s at t/T = 0.75 respectively. Average velocity magnitude in the left and right maxillary ostium region was 0.15 m/s and 0.41 m/s at t/T = 0.25; 0.16 m/s and 0.4 m/s respectively at t/T = 0.75, respectively. Hence similar average flow was observed between inspiration and expiration.

Flow at maxillary and frontal ostium in pre-operative subject-2A are zero or near to zero and not discussed. In comparison, surgery significantly altered flow characteristics at maxillary ostium. For subject-2B, maximum velocity at left and right maxillary ostium were 0.33 m/s and 1.53 m/s at t/T = 0.25; 0.1 m/s and 0.16 m/s at t/T = 0.75 respectively. Average velocity at left and right maxillary ostium was 0.08 m/s and 0.34 m/s at t/T = 0.25; and 0.02 m/s and 0.05 m/s at t/T = 0.75, respectively. In subject-3, maximum velocity at left and right maxillary ostium was 0.59 m/s and 0.44 m/s at t/T = 0.25; and 0.43 m/s and 0.24 m/s at t/T = 0.75, respectively. Average velocity at left and right maxillary ostium was 0.29 m/s and 0.21 m/s at t/T = 0.25; and 0.11 m/s and 0.08 m/s at t/T = 0.75, respectively. In the post-operative subjects, velocity at the maxillary ostia was significantly greater during inspiration than expiration mainly attributed to large size of maxillary ostium resulting from the antrostomy.

Airflow at frontal ostium was also recorded. For subject-1, average velocity observed at the left and right frontal ostium is 0.06 m/s and 0.02 m/s at t/T = 0.25; and 0.07 m/s and 0.03 m/s at t/T = 0.75, respectively. Furthermore, inside the frontal sinus airflow was much smaller than 0.01 m/s. As before, we do not discuss subject-2A as airflow speed at frontal ostium was near zero. In subject-2B, maximum velocity at left and right frontal ostium was 0.002 m/s and 0.03 m/s at t/T = 0.25; and 0.15 m/s and 0.07 m/s respectively at t/T = 0.75. Average velocity at left and right frontal ostium was 0.0006 m/s and 0.006 m/s at t/T = 0.25; and 0.03 m/s and 0.01 m/s at t/T = 0.75, respectively. Hence surgery has increased the average flow speed at the frontal ostium during expiration compared to inspiration. Unlike subject-2b, one would expect prominent effects of drillout surgery on flow near the frontal sinus region. Accordingly, in subject-3 maximum velocity in frontal ostium was 2.06 m/s at t/T = 0.25; and 1.3 m/s at t/T = 0.75. Average velocity was 0.5 m/s at t/T = 0.25 respectively and 0.3 m/s at t/T = 0.75.

In subject-3, velocity at preselected locations in the frontal and maxillary ostium are shown in supplementary material (S6 Fig). Spike-like oscillations were observed symbolic of flow instability. The mechanism behind origin of this self-sustained oscillations is not known. Such instability have been previously observed in open cavity flows in both laminar and turbulent flow settings. Self-sustained oscillations [34] were demonstrated in certain regimes of Reynolds numbers and cavity shape. For comparison, we also performed another simulation with peak flow rate of 6 L/min and time period of T = 4 seconds for subject-c only. This flow rate is smaller than conventionally assumed for quiet breathing. Nevertheless, these spike-like oscillations in velocity were not present at the ostia (see S6 Fig). Further investigations on mechanism of these oscillations are beyond the scope of this work.

Airflow streamlines for normal and post-operative airways are provided in supplementary material. The streamlines in subject-c was of special interest as it showed interesting behavior due to the common bore at the septum. Fig 11 shows instantaneous streamlines near the frontal ostium for the drill-out subject whose frontal ostium is a ‘U-shaped’ section. During inspiration, streamlines mostly enter the frontal sinus through the right nose due to the notched shape of nasal vestibule: streamlines splits due to the common bore in the septum and enters the frontal sinus. Streamlines exit the frontal recess in a complex fashion circulating into the ethmoidal sinuses and eventually flow out of the nasopharyngeal region. During expiration, airflow streamlines enter the frontal opening mostly from the right nasal airway. Due to the shape of the nasal passage around the ostium, streamlines enter through the anterior section as seen from vector plot Fig 11(b). On leaving the frontal opening, streamlines split again (due to the common drainage pathway through the nasal septum) and flow out through the left and right nose as indicated by the black solid arrows in Fig 11(b). In summary, air ventilates the frontal sinuses differently between inspiration and expiration: may have a preferred nasal side through which entrainment occurs and is an effect of frontal surgery and nasal vestibule notch.

## Discussion

The fluid dynamic computations in this study have provided insight into the effects of anatomy and surgery on sinonasal ventilation. In subject-2, nasal resistance was reduced after FESS. Surgery significantly altered flow characteristics at the maxillary ostium. Compared to the healthy normal subject, significant asymmetry in airflow between inspiration and expiration was introduced by surgery. In the post-operative subject-2b, inspiration velocity differed (from exhalation) by upto ten times at the maxillary ostium. During FESS, the removal of polyps, inflammatory tissue and changing anatomy of the frontal sinus is often limited by the frontal beak [35, 36] and proximity to brain and eye. Hence patency of the frontal ostium was greatly improved but still FESS has not greatly increased ventilation into frontal sinus. On the other hand, the result of drillout procedure on frontal ostium region is more extensive. The frontal beak, frontal intersinus septum and the adjacent nasal septum are removed, creating airflow pathway into and out of the frontal sinus. Hence frontal sinus-nasal passage interaction is more prominent in subject-3 than subject-2b. One highlight was difference in maximum and average velocity between left and right nasal airway. Sometimes the difference is significant as in the case of maxillary ostium in subject-2b. Hence such differences between left and right airway must be accounted to study nasal airflow using unilateral airway models compared to bilateral airway models.

One highlight of this study was use of the same subjects’ pre-operative and post-operative nasal airway geometry. We acknowledge that only one healthy normal has been used in this study. Human airway geometry presents variations in nostril, valve and meati sizes and shapes. Our healthy normal airway showed features matching with observations reported previously such as greater flow through common meatus, small pressure drop across maxillary sinus, mostly straight streamtraces in the lower part of cavity from anterior to posterior cavity. Xiong et al [12] estimated flow in different coronal planes in their simulation. They observed pressure difference of 0.09 Pa (during inhalation) and 0.03 Pa (during exhalation) between maxillary opening and maxillary sinus. We observed 0.04 Pa (at peak inhalation) and 0.01 Pa (at peak exhalation) which agrees well. Wen et al. [37] reported flow distribution in different sub-sections of a turbinate section for 15L/min flow rate. In the right airway, flow was computed in the middle medial airway as 28.8%, 23.7% in the superior medial section and 2.1% in the inferior meatus extension. In subject-1 at 12 L/min, we computed 20%, 10% and 3% flow rate distribution in these three regions, respectively. Quantitative differences are to be expected among individual subjects across available literature. Nevertheless, comparing normal with post-operative environments are a useful way to understand effects of surgery beyond the obvious betterment in nasal congestion.

The characteristics of airflow at the ostium and within the sinuses are analogous to an open cavity flow. Open cavity are attached to external channel or duct. Incompressible flows over cavities have been widely studied [38–41]. At low Reynolds numbers, the flow in the cavity is separated from outer ductal flow. At higher Reynolds number or presence of alternating pressure gradient in the duct, streamlines enter the cavity permitting transport into and out of the cavity. Similar insights into sinonasal mass transport [40, 42] could be gained from considering equivalent open cavity geometry.

## Study limitations

Results from the current study point to the need for a case-by-case study of changes to airflow following surgery. This study also has highlighted the need for a bilateral airway model in post-surgical patients. This study has relied on the geometry of a small number of patients in a clinical spectrum between asymptomatic and surgically treated symptomatic CRS. Segmentation and development of models suitable for airflow studies relies on accurate representation of bony cavities. Patients with CRS typically have soft tissue, mucosal inflammation and swelling which can underestimate the size of cavities and ostia. Patients who improve after surgery are usually not exposed to further scans and radiation. For this reason, large patient study numbers would be difficult to acquire. More recently, virtual surgery is chosen as an alternative to investigate airflow in post-operative subjects. In our case, post-operative cases were patients who were symptomatic and warranted a further investigation, but CT-scan appearances proved to be without ongoing inflammatory changes. We believe that although there are anatomic variations between individuals, these studies accurately depict the spectrum of clinical presentation and geometry.

## Supporting Information

S1 Fig Instantaneous streamline plot at peak inspiration (t/T = 0.25) and peak expiration (t/T = 0.75) in the healthy normal subject-1. (JPG)

S2 Fig Instantaneous streamline plot at peak inspiration (t/T = 0.25) and peak expiration (t/T = 0.75) near left maxillary ostium for the post-operative subject-2b. (JPG)

S3 Fig Instantaneous streamline plot at peak inspiration (t/T = 0.25) and peak expiration (t/T = 0.75) near maxillary ostium for the drillout subject-3. (JPG)

S4 Fig Time history of velocity measured at maxillary and frontal ostium for healthy normal subject-1. (JPG)

S5 Fig Time history of velocity measured at maxillary and frontal ostium for post-operative subject-2b. (JPG)

S6 Fig Time history of velocity measured at maxillary and frontal ostium for drillout subject-3 for 12L/min and 6L/min. (JPG)
