# Supplementary figures and images for: Airflow in the Human Nasal Passage and Sinuses of Chronic Rhinosinusitis Subjects
Source: PLoS One. 2016 Jun 1;11(6):e0156379. doi: 10.1371/journal.pone.0156379 (PMC4889048; doi:10.1371/journal.pone.0156379)

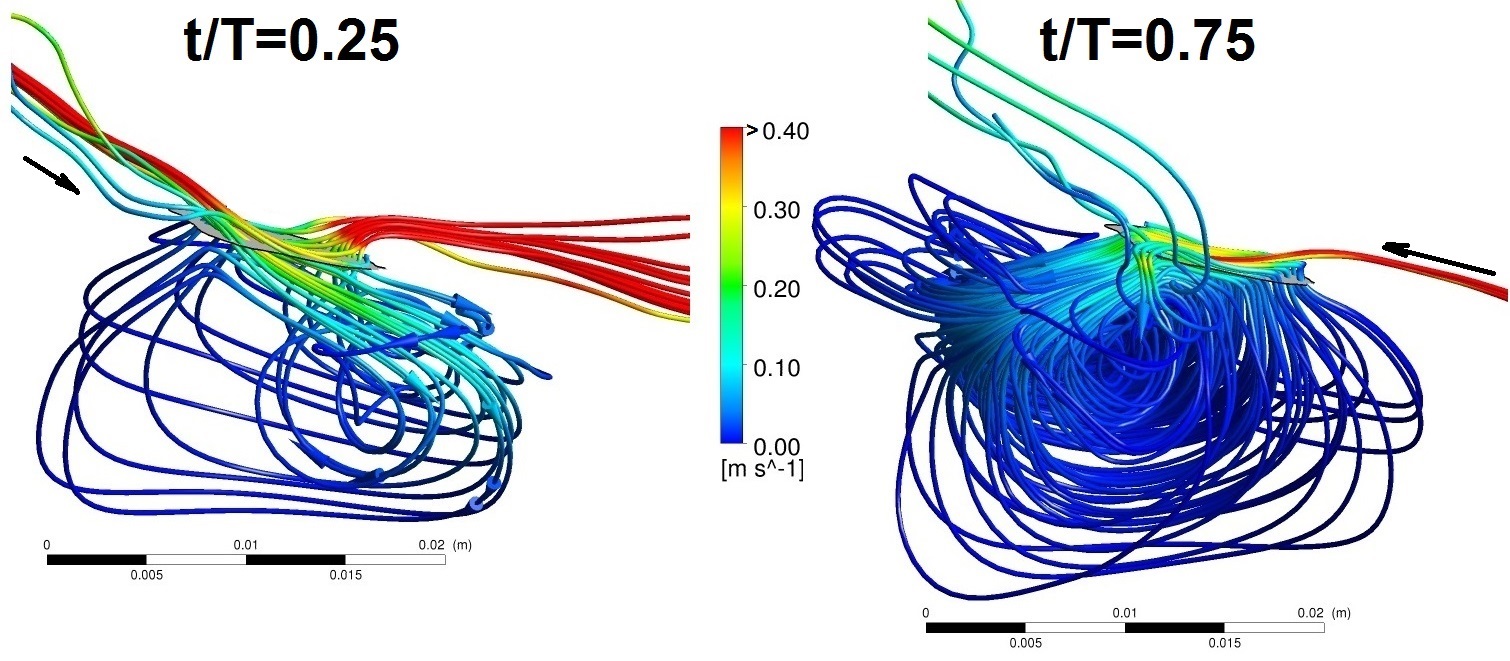

Supplement: S1 Fig — (JPG) [file pone.0156379.s001.jpg]

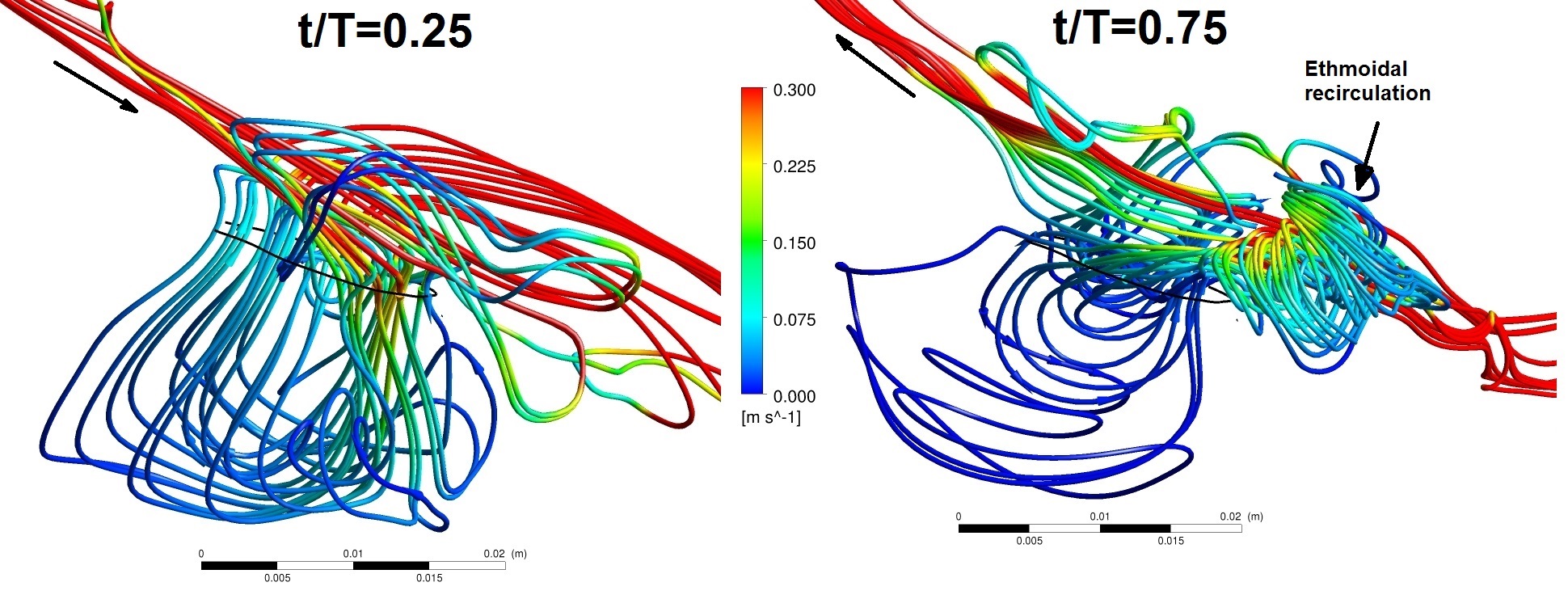

Supplement: S2 Fig — (JPG) [file pone.0156379.s002.jpg]

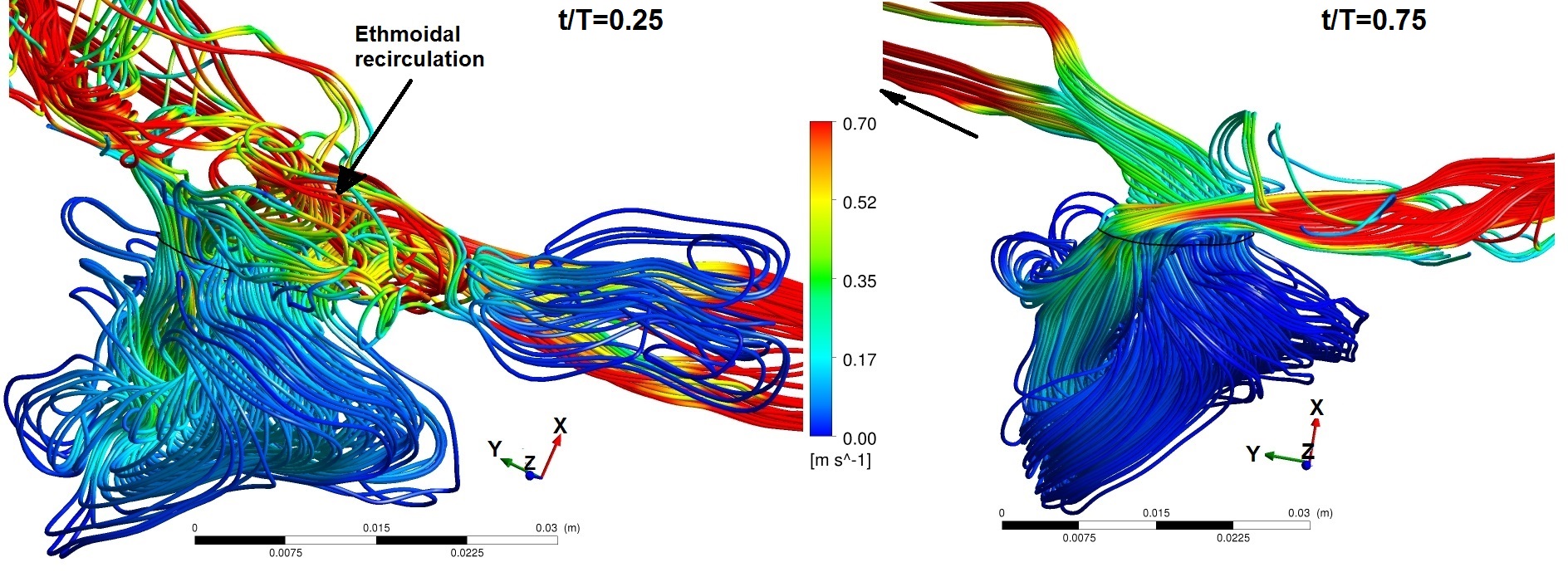

Supplement: S3 Fig — (JPG) [file pone.0156379.s003.jpg]

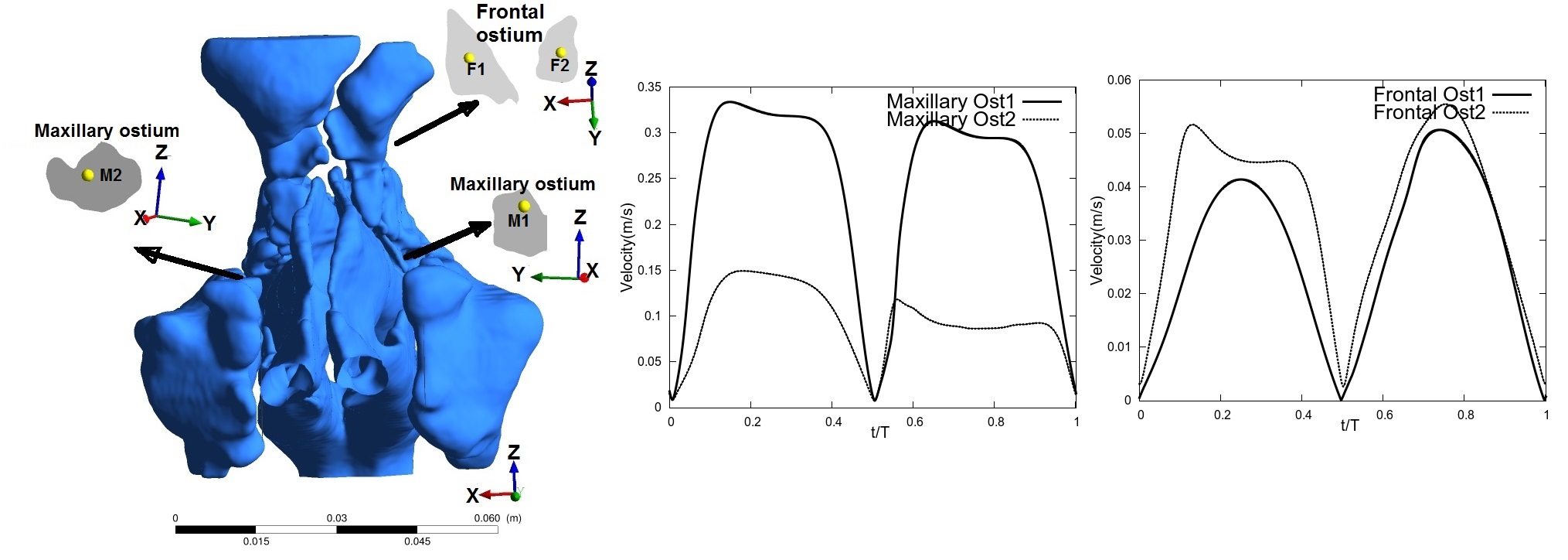

Supplement: S4 Fig — (JPG) [file pone.0156379.s004.jpg]

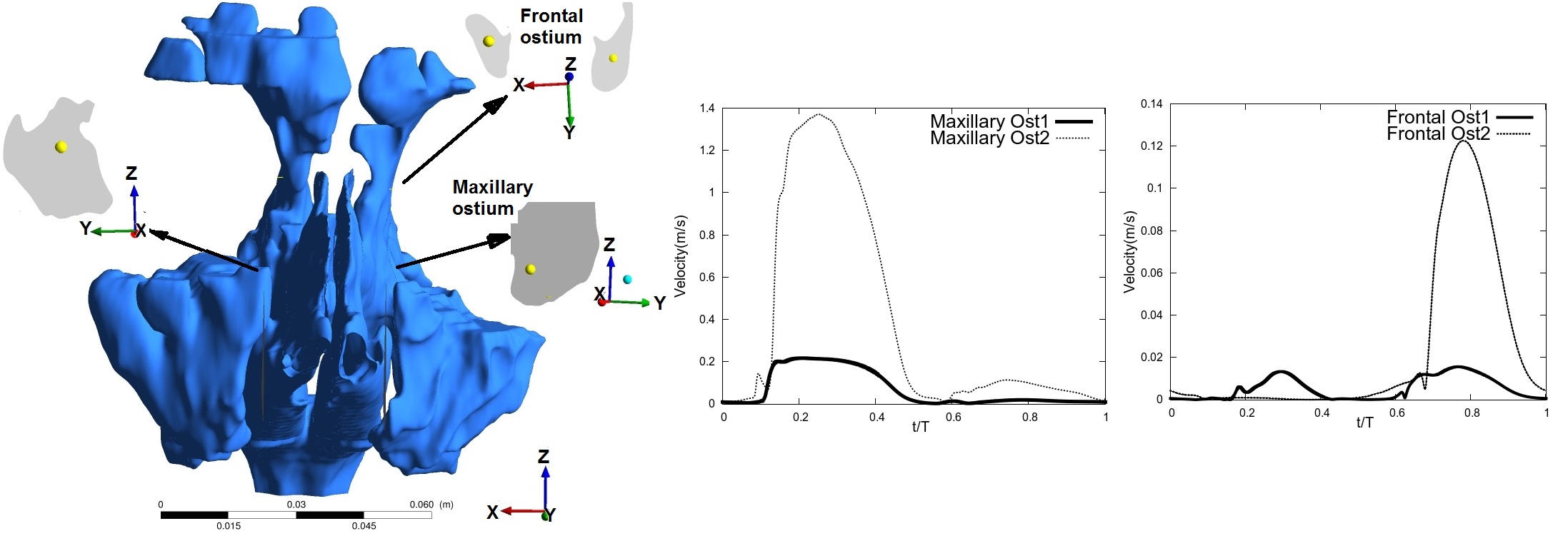

Supplement: S5 Fig — (JPG) [file pone.0156379.s005.jpg]

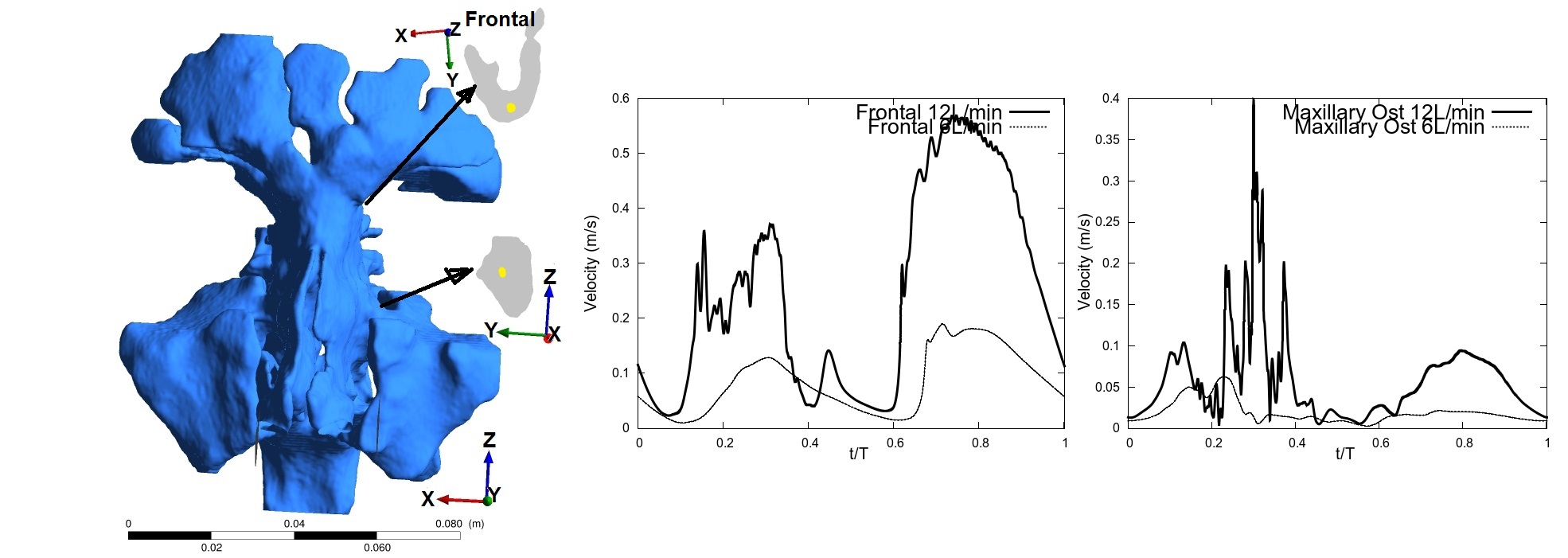

Supplement: S6 Fig — (JPG) [file pone.0156379.s006.jpg]
